# Application of Glass Fiber and Carbon Fiber-Reinforced Thermoplastics in Face Guards

**DOI:** 10.3390/polym13010018

**Published:** 2020-12-23

**Authors:** Takahiro Wada, Hiroshi Churei, Mako Yokose, Naohiko Iwasaki, Hidekazu Takahashi, Motohiro Uo

**Affiliations:** 1 Department of Advanced Biomaterials, Graduate School of Medical and Dental Sciences, Tokyo Medical and Dental University, 1-5-45 Yushima, Bunkyo-ku, Tokyo 113-8549, Japan; yokose.peri@tmd.ac.jp (M.Y.); uo.abm@tmd.ac.jp (M.U.); 2 Department of Sports Medicine/Dentistry, Graduate School of Medical and Dental Sciences, Tokyo Medical and Dental University, 1-5-45 Yushima, Bunkyo-ku, Tokyo 113-8549, Japan; chu.spmd@tmd.ac.jp; 3 Department of Oral Biomaterials Development Engineering, Graduate School of Medical and Dental Sciences, Tokyo Medical and Dental University, 1-5-45 Yushima, Bunkyo-ku, Tokyo 113-8549, Japan; iwasaki.bmoe@tmd.ac.jp (N.I.); takahashi.bmoe@tmd.ac.jp (H.T.); 4 Department of Materials Engineering, Graduate School of Engineering, The University of Tokyo, 7‑3‑1 Hongo, Bunkyo‑ku, Tokyo 113-8656, Japan

**Keywords:** face guard, glass fiber-reinforced thermoplastic, carbon fiber-reinforced thermoplastic, shock absorption

## Abstract

Face guards (FGs) are protectors that allow for the rapid and safe return of athletes who are to play after sustaining traumatic facial injuries and orbital fractures. Current FGs require significant thickness to achieve sufficient shock absorption abilities. However, their weight and thickness render the FGs uncomfortable and reduce the field of vision of the athlete, thus hindering their performance. Therefore, thin and lightweight FGs are required. We fabricated FGs using commercial glass fiber-reinforced thermoplastic (GFRTP) and carbon fiber-reinforced thermoplastic (CFRTP) resins to achieve these requirements and investigated their shock absorption abilities through impact testing. The results showed that an FG composed of CFRTP is thinner and lighter than a conventional FG and has sufficient shock absorption ability. The fabrication method of an FG comprising CFRTP is similar to the conventional method. FGs composed of commercial FRTPs exhibit adequate shock absorption abilities and are thinner and lower in weight as compared to conventional FGs.

## 1. Introduction

In contact sports such as soccer, rugby, and American football, maxillofacial traumatic injuries can occur [1,2,3,4,5,6,7,8]. A face guard (FG) is a piece of protective equipment worn by an athlete suffering a maxillofacial injury and allows for the earlier and safer return of the injured athlete to the sport. The effectiveness of FGs has been widely recognized [9,10,11,12,13,14,15,16,17,18]. An FG must fulfill the following three requirements: (i) protect the player from re-injury (protection ability), (ii) avoid injury to other players (safety), and (iii) avoid narrowing the player’s field of vision (maintain performance) [16]. In particular, the first two requirements are stated in the *Laws of the Game* by the International Football Association Board as follows: “Non-dangerous protective equipment, for example headgear, facemasks and knee and arm protectors made of soft, lightweight padded material is permitted as are goalkeepers’ caps and sports spectacles [19].” Moreover, any effect on the field of vision must be minimized to maintain the performance (requirement (iii)), which is illustrated by the clinical effectiveness of the FG design based on objective data from visual field tests [20].

The players who used conventional FGs were satisfied with the protective ability of the FG (requirements (i) and (ii)) but were dissatisfied with the comfort, claiming that it slipped off while playing and narrowed their field of vision. Therefore, thinner and lighter FGs are required, especially for professional players who are concerned by the FG’s bulkiness and the reduction of their field of vision [15,20].

A conventional FG is made of a core material and a cushioning material that covers the inner and outer layers of the core material. The core material is often a thermoplastic resin, whose molding temperature is a key factor in the FG fabrication process. Industrial thermoplastic resins are usually formed using a non-customizable mold with sufficient mechanical strength at high working temperatures and forming pressures. However, FG fabrication should be carried out using a plaster mold of the patient’s face that can be prepared without the use of expensive equipment. Therefore, there is a need to find an alternative to the above forming process that uses lower temperatures and pressures. The materials used in this alternative process should be easily molded using manual pressure and hot water (above 60 °C) or a low power hot plate [12,15,21,22]. However, thermoplastic resins with low molding temperatures have relatively poor mechanical properties; therefore, FGs made with these thermoplastic resins generally need to be thicker than those made with a thermoset resin [23].

To solve this problem, Abe et al. [23] attempted to reduce the thickness of the core material of an FG by reinforcing the conventional thermoplastic material with fiberglass, widely known as fiber-reinforced thermoplastic (FRTP) materials. They reported that FGs made of FRTPs have remarkable shock absorption abilities and were thinner than a conventional FG, with a decrease of 1.7 mm in thickness. We researched FGs that incorporated a glass fiber-reinforced thermoplastic (GFRTP) and a buffering space [24]. The design adopted in this method requires a 50 mm square of GFRTP, which sufficiently covers the buffering space (30 mm in diameter). The amount of GFRTP required could be minimized; however, in this study, there were problems that made it necessary to make our own GFRTP.

In recent years, FRTPs have attracted attention in many fields, such as automotive, sports, and construction industries, due to their low weight, high flexural modulus, and good workability. The increased demand for FRTPs has led to increased availability and variety at lower costs. Some of these FRTPs can be molded at lower temperatures and pressures.

If commercially available FRTPs can be used in FGs, we can make lightweight and thin FGs without a special fabrication process. However, the effects of the use of FRTPs on the shock absorption ability, weight reduction, and thinness of FRTP-based FGs have not been investigated. In this study, trial FGs made of commercial FRTPs were fabricated to determine the effects of material type on the prevention ability, weight reduction, and thinness of the FGs.

## 2. Materials and Methods

### 2.1. Materials

Aquaplast (AP; Homecraft Rolyan, Huthwaite, North Nottingham, UK) specimens with a thicknesses of 3.2 mm (AP32) and 1.6 mm (AP16) were selected, as they are commercial thermoplastic resins commonly used in medical splints. Two commercial FRTP materials with a thickness of 1.0 mm, GFRTP (Tepex dynalite108, Bond Laminates GmbH, Brilon, Germany) and carbon fiber-reinforced thermoplastics (CFRTP; Tepex dynalite208, Bond Laminates GmbH, Brilon, Germany) were examined for their suitability as FG core materials (Table 1).

### 2.2. Three-Point Bending Test

Three-point bending tests were performed according to Japanese Industrial Standards (JIS) K7171-2008 [25] and K7074-1988 [26] using a universal testing machine (EZ-LX, Shimadzu Co., Tokyo, Japan). The specimens for the three-point bending test were prepared using an ultrasonic cutter and measured using a micrometer (293-421-20; Mitsutoyo, Kanagawa, Japan; minimum reading: 0.001 mm). The flexural strength and flexural modulus were calculated by the following equations using TRAPEZIUM X ver. 1.4.0 analysis software (Shimadzu Co., Tokyo, Japan).

Flexural strength = 3Fl/(2bh^2^), (1)

Flexural modulus = (F_1_l^3^)/(4bh^3^d), (2)

Here, F is the maximum load (N), l is the width of the support span (mm), b is the width (mm) of the specimen, h is the height (mm) of the specimen, F1 is the load (N) at a point in the linear portion of the trace, and d is the deflection (mm) at load F1. Five specimens were examined for each material.

### 2.3. Shock Absorption Test

The core materials (100 mm × 100 mm in size) used for the shock absorption tests were prepared using an ultrasonic cutter. To simulate real-world use, the core materials were covered with a cushioning material (Neoprene, Homecraft Rolyan, Huthwaite, North Nottingham, UK) on both sides using a 2 g cyanoacrylate adhesive (Aron Alpha #35045, Konishi Co., Osaka, Japan) 24 h prior to the measurement. The cushioning materials are necessary for the protection of the wearer and other players.

Shock absorption tests were carried out using an impact testing machine (modified IM-201, Tester Sangyo Co., Saitama, Japan). The impact was applied to the specimens by a 500 g weight dropped from a height of 240 mm onto a steel rod positioned directly above the sample with a 6.34 mm diameter rounded end. Two measuring systems were used: a load cell and a pressure measurement film.

#### 2.3.1. Time Change of the Impact Load

The impact load was measured by three dynamic compression load cells (LMB-A-2KN, Kyowa Electronic Instruments Co., Tokyo, Japan) that were placed in a triangular formation below a 10 mm thick stainless steel platform supporting the specimen. The specimen was placed at the center of the platform. When impact was applied, the load was recorded by a universal recorder (EDX-100A, Kyowa Electronic Instruments Co., Ltd., Tokyo, Japan) using DCS-100A data acquisition software (Kyowa Electronic Instruments Co., Ltd., Tokyo, Japan) at a sampling rate of 20 kHz. The total impacted load was calculated as the sum of the loads recorded by the three load cells. For a reference measurement, the total impacted load was measured without a specimen.

#### 2.3.2. Impact Pressure Distribution

The impact pressure distribution below the FG was estimated using a pressure measurement film (Presheet, Fujifilm Corp., Tokyo, Japan) that was placed under the specimen. Films with two different sensitivities (covering pressure ranges of 2.5–10.0 MPa (LW) and 0.5–2.5 MPa (LLW)) were used. The pressed region of the film exhibited red coloration depending on the pressure. The pressure distribution and maximum pressure were analyzed using Data Shot FPD-100S ver. 1.0 image analysis software (Fujifilm Corp., Tokyo, Japan; ImageJ ver. 1.47t; National Institutes of Health (NIH), Bethesda, MD, USA [27]). Five impact loads were applied to each specimen, and five specimens were examined for each set of conditions.

### 2.4. Statistical Analysis

The obtained results were analyzed using a one-way analysis of variance with Tukey’s honestly significant difference test, using JMP ver. 13.2.1 statistical software (SAS Institute Inc., Cary, NC, USA) with a significance level of 5%.

## 3. Results

### 3.1. Flexural Strength and Modulus

**Figure 1:**
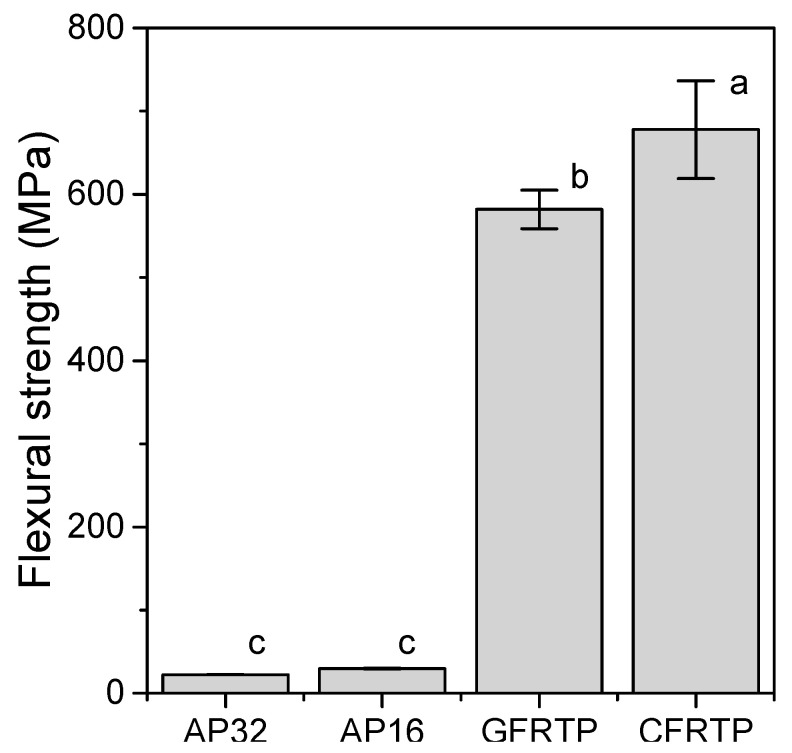
Flexural strengths of AP32, AP16, GFRTP, and CFRTP. Bars labeled with the same letter show no significant difference (*p* > 0.05).

**Figure 2:**
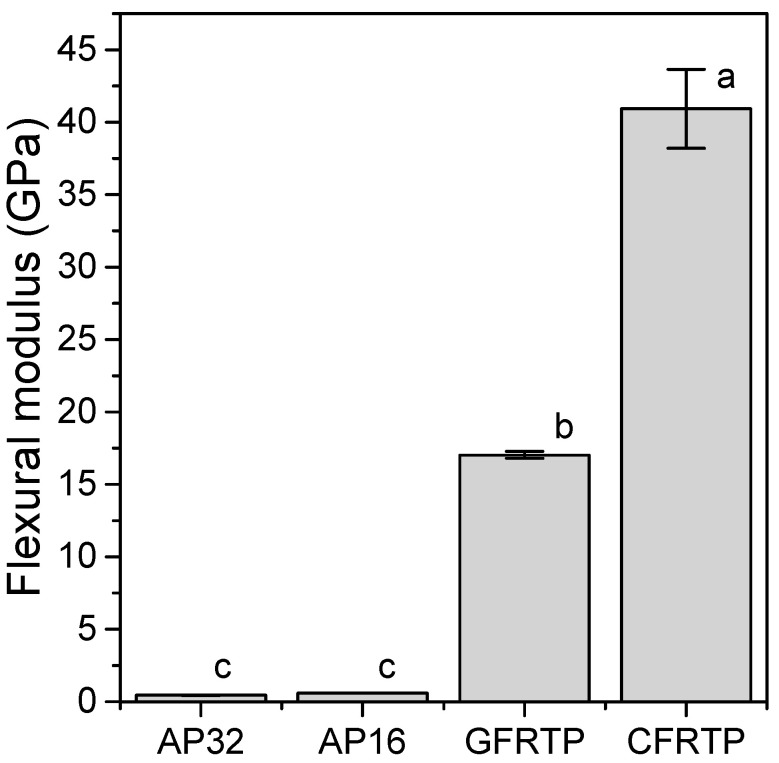
Flexural moduli of AP32, AP16, GFRTP, and CFRTP. Bars labeled with the same letter show no significant difference (*p* > 0.05).

Figure 1 shows the flexural strength calculated from the three-point bending test. None of the specimens fractured during the experiment. The flexural strengths of the FRTPs (GFRTP: 582 ± 23 MPa, CFRTP: 678 ± 59 MPa) were greater than those of AP (c.a. 26 MPa (specifically, AP32: 22.2 ± 0.2 MPa, AP16: 29.4 ± 0.6 MPa)). The flexural moduli obtained from the three-point bending tests are shown in Figure 2. The flexural moduli of the FRTPs (GFRTP: 17.0 ± 0.3 MPa, CFRTP: 40.9 ± 2.7 GPa) were significantly greater than those of AP (c.a. 0.5 GPa (AP32: 0.44 ± 0.23 GPa, AP16: 0.60 ± 0.08 GPa)) and other commercial thermoplastic resins used to prepare medical splints (0.47–2.25 GPa) [28].

### 3.2. Time Change of the Impact Load

**Figure 3:**
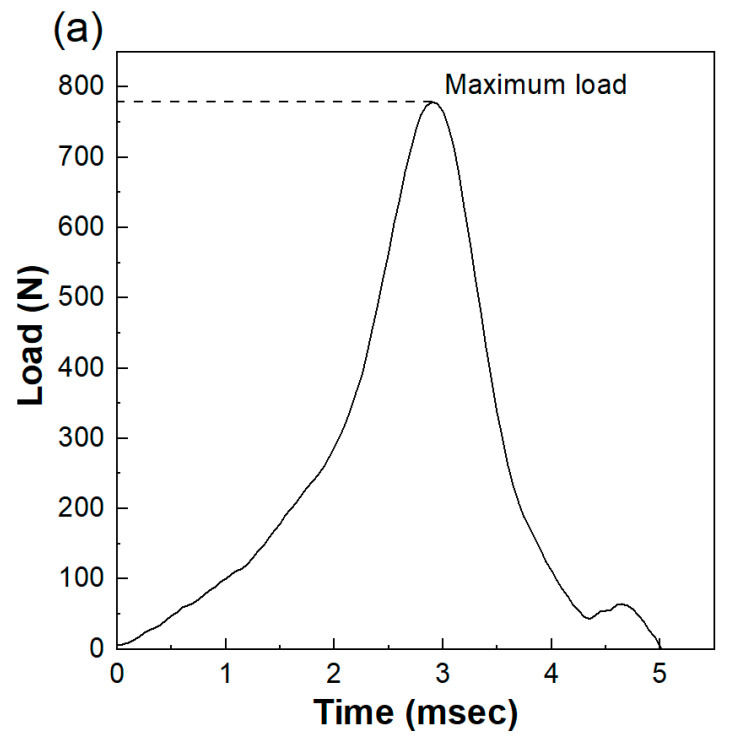

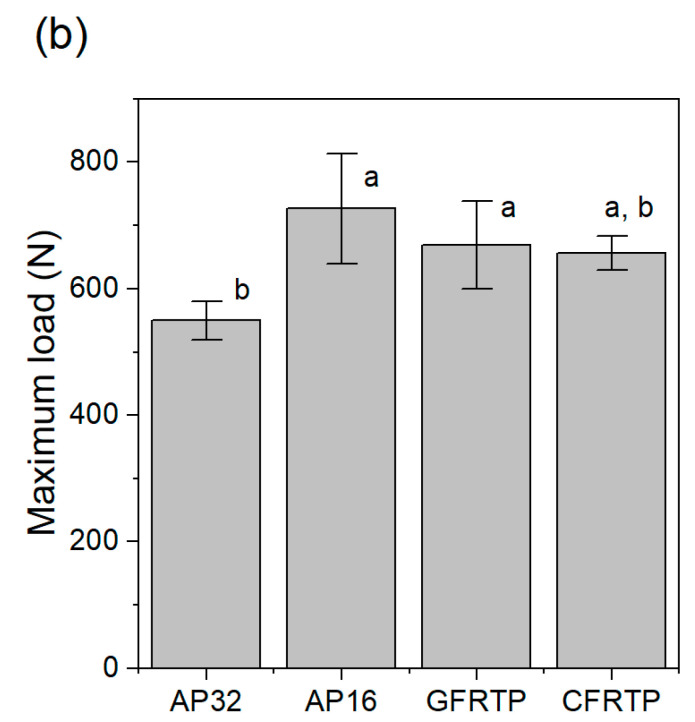
(**a**) Load obtained from shock absorption of AP16. (**b**) Maximum loads of AP32, AP16, GFRTP, and CFRTP. Bars labeled with the same letter show no significant difference (*p* > 0.05).

The maximum load of the impact test without a specimen was 5209 ± 293 N. As shown in Figure 3a,b the maximum load decreased in the presence of a specimen. The maximum loads of AP32 and AP16 were 550 ± 30 N and 727 ± 87 N, respectively, while the maximum load of GFRTP (669 ± 70 N) was slightly higher than that of AP32. The maximum load of CFRTP (656 ± 27 N) was not significantly different from that of AP32 (*p* < 0.05).

### 3.3. Pressure Distribution under the FG

**Figure 4:**
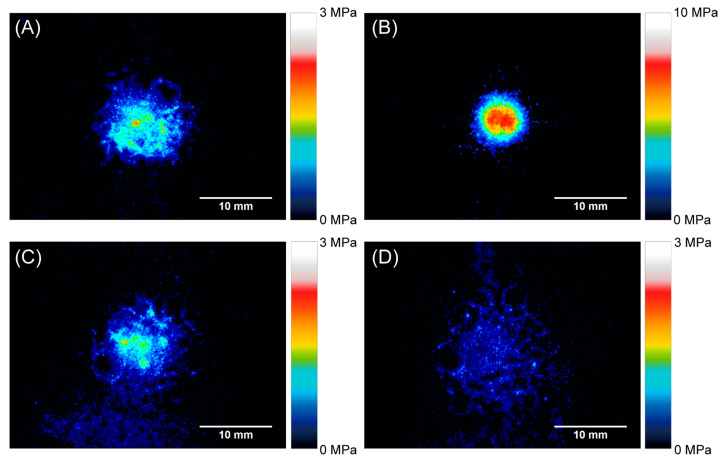
Pressure distribution obtained using pressure measurement films. (**A**) AP32, (**B**) AP16, (**C**) GFRTP, and (**D**) CFRTP.

**Figure 5:**
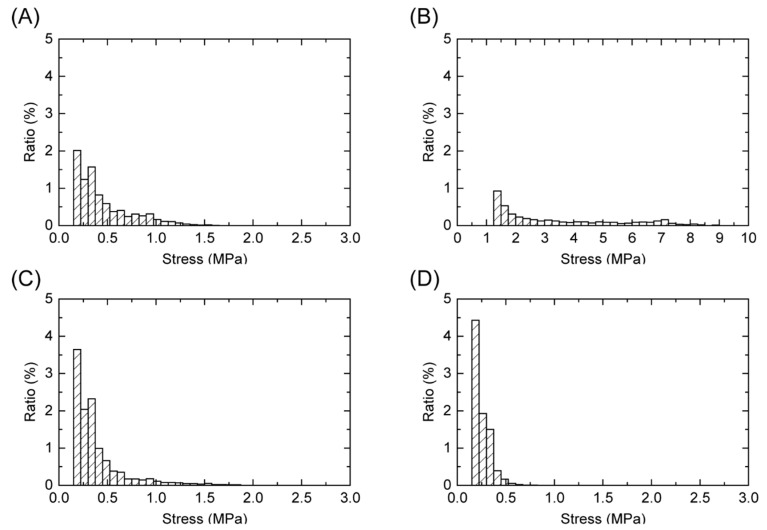
Pressure histograms obtained using pressure measurement films. (**A**) AP32, (**B**) AP16, (**C**) GFRTP, and (**D**) CFRTP.

**Figure 6:**
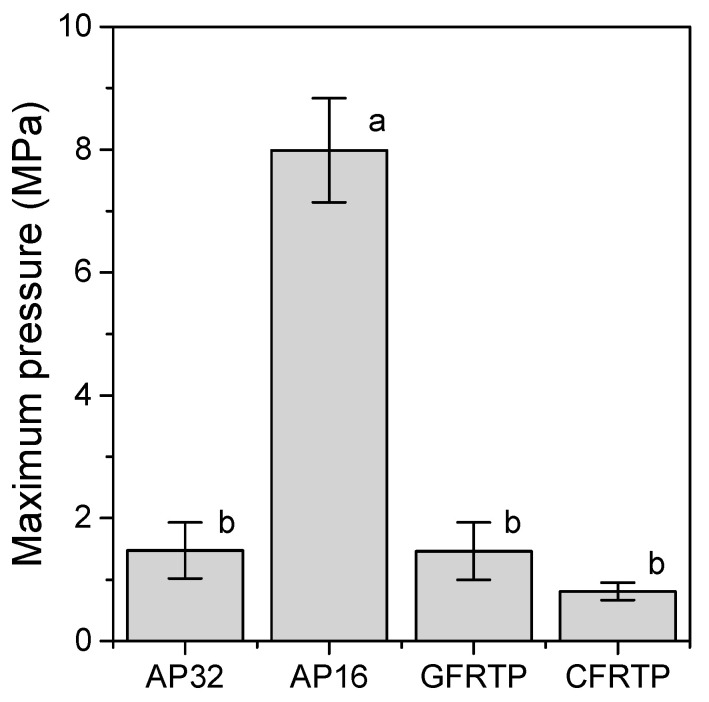
Maximum pressure obtained from shock absorption tests. Bars labeled with the same letter show no significant difference (*p* > 0.05).

Figure 4 and Figure 5 show the pressure distributions and histograms measured using pressure measurement films in the shock absorption tests. The site of impact on AP16 can be clearly seen in Figure 4b, and this material had the largest area with the highest pressure of all tested materials. In the cases of AP32, GFRTP, and CFRTP, although the results of these are in the same pressure range, the CFRTP sufficiently dispersed the impact. The histograms in Figure 5 also show the pressure distribution of the FRTPs shifted to a lower pressure range. These results show that FRTPs can be used to effectively disperse impacts. The maximum pressures were also analyzed using the pressure measurement film, as shown in Figure 6. The maximum pressure of AP16 (7.99 ± 0.85 MPa) was the highest, whereas the maximum pressure of CFRTP was the lowest (0.81 ± 0.14 MPa). The maximum pressures of AP32 and GFRTP were 1.48 ± 0.45 and 1.46 ± 0.47 MPa, respectively.

## 4. Discussion

The three-point bending test was used to determine the flexural strength and flexural modulus. The flexural strength is the maximum pressure achieved during the test, which indicates the amount of pressure that can be applied before fracture. The flexural modulus is the constant of deflection of the material, which represents the bendability of the material within the elastic deformation limits. The GFRTP and CFRTP materials showed greater flexural strength and flexural modulus. A core material with a higher flexural module is expected to diffuse impacts more effectively.

The load cell system used in the present study could monitor the load transmitted below the FG materials over time; therefore, the degree of change in the impact load was recorded. The impact force required to fracture the human maxillofacial bone has been reported to be between 4930 and 5780 N [29]; therefore, the impact load of a free-falling weight (500 g) from a 240 mm height (5209 ± 293 N) was used in the present study. The impact load absorption capability, which is the ratio of the decreased impact load by the FG material to the original impact load, has often been discussed [29,30,31]. Previous research, in which an impact load system similar to that of the present study was used, reported that the impact load absorption capabilities of the medical splint materials ranged from 85% to 88% [30]. These results are consistent with the results of the present study (86% to 89%). The impact load absorption capabilities of GFRTP and CFRTP are 87%, which is the same as those of the medical splint materials.

The pressure measurement film can precisely record the impact area shown by a change in color from blue to red [32]. From this color density, the level of pressure can be analyzed using a digital camera or scanner and analysis software. Each pressure measurement film has a limited range of sensitivity; therefore, LW and LLW-type pressure measurement films were used in this study. However, the areas where the impact pressure was lower than 0.5 MPa and higher than 10 MPa could not be detected. As a result, the maximum pressures were measured for all of the specimens except for the reference. Generally, the specimen with a lower maximum pressure had a smaller impressed area. FG materials with lower maximum pressures are preferable for protecting the injured area. Therefore, AP32, GFRTP, and CFRTP are more suitable than AP16. The maximum pressures of AP32 and GFRTP (1.48 ± 0.45 MPa and 1.46 ± 0.47 MPa, respectively) are higher than that of CFRTP (0.81 ± 0.14 MPa). This suggests that AP32 and GFRTP can bend more than CFRTP, as AP and GFRTP are more flexible than CFRTP. A decrease in maximum pressure implies a good dispersion of impact. These results suggest that CFRTP possesses suitable shock absorption properties for application in FGs despite being relatively thin.

The weight of the CFRTP sample (34 g) without the cushioning material was approximately 40% lower than that of the conventional FG (56 g). The thickness of the CFRTP sample (1 mm) without the cushioning material was 2.2 mm less than that of the conventional FG (3.2 mm). Therefore, the shock absorption property of the experimental FG made with CFRTP would adequately protect the injured players from impact while improving their field of vision and the comfort of the FG when compared to the conventional FGs.

Reinforcement by glass or carbon fibers can effectively provide thin and light FGs with enough shock absorption ability. The thickness and weight of the core materials used in this study are approximately one-third to one-half of those of AP32. Core materials are crucial for the dispersion and absorption of impact. The results of this study showed that the 1 mm thick CFRTP is a better choice than the 1 mm thick GFRTP.

The preparation of CFRTP is neither time-consuming nor expensive as compared with AP. CFRTPs can be prepared by a conventional method. The molding temperature of CFRTP used in this study was 220 °C, which is higher than that of AP (a molding temperature of 75 °C). CFRTP cannot be molded using hot water like AP, but it can mold by heating the material to 220 °C using a low-power hot plate. Recently, the demand for GFRTP and CFRTP has resulted in a lower price. Therefore, the additional cost of using this method, the material cost, and the preparation time are almost the same.

In this study, we researched only one type of FRTP, GFRTP, and CFRTP with a thickness of 1 mm and demonstrated only one potential of an FRTP. In the future, we will optimize FRTPs for FGs in terms of types of fibers and matrices, their relative volumes, and thicknesses.

## 5. Conclusions

In this study, we prepared FGs made of commercial FRTPs. A comparison of their shock absorption ability with those of conventional specimens revealed that they have the same level of shock absorption ability but a lower thickness and weight. Commercial FRTP can be used as a material for FGs in practical applications, although alternative products need to be considered.

## Figures and Tables

**Table 1:** Specifications of the materials used.

Code	Composition	Fiber (Fiber Volume Content)	Density Thickness (g/cm^2^)	Thickness (mm)	Weight of Specimen (g)
AP32	Polycaprolactone	-	0.35	3.2	55.6
AP16	Polycaprolactone	-	0.19	1.6	39.1
GFRTP	Thermoplastic Polyurethane	Glass (45 vol.%)	0.18	1.0	36.9
CFRTP	Thermoplastic Polyurethane	Carbon (45 vol.%)	0.15	1.0	34.2

## Data Availability

The data that support the findings of this study are available from the corresponding author (T.W.) upon reasonable request.
